# Predicting Functional Recovery after Acute Ankle Sprain

**DOI:** 10.1371/journal.pone.0072124

**Published:** 2013-08-05

**Authors:** Sean R. O’Connor, Chris M. Bleakley, Mark A. Tully, Suzanne M. McDonough

**Affiliations:** 1 Faculty of Science and Engineering, University of Brighton, Brighton, United Kingdom; 2 Faculty of Life and Health Science, University of Ulster, Belfast, Northern Ireland, United Kingdom; 3 UKCRC Centre of Excellence for Public Health (NI), Queen’s University Belfast, Belfast, Northern Ireland, United Kingdom; The University of Queensland, Australia

## Abstract

**Introduction:**

Ankle sprains are among the most common acute musculoskeletal conditions presenting to primary care. Their clinical course is variable but there are limited recommendations on prognostic factors. Our primary aim was to identify clinical predictors of short and medium term functional recovery after ankle sprain.

**Methods:**

A secondary analysis of data from adult participants (N = 85) with an acute ankle sprain, enrolled in a randomized controlled trial was undertaken. The predictive value of variables (age, BMI, gender, injury mechanism, previous injury, weight-bearing status, medial joint line pain, pain during weight-bearing dorsiflexion and lateral hop test) recorded at baseline and at 4 weeks post injury were investigated for their prognostic ability. Recovery was determined from measures of subjective ankle function at short (4 weeks) and medium term (4 months) follow ups. Multivariate stepwise linear regression analyses were undertaken to evaluate the association between the aforementioned variables and functional recovery.

**Results:**

Greater age, greater injury grade and weight-bearing status at baseline were associated with lower function at 4 weeks post injury (p<0.01; adjusted R square=0.34). Greater age, weight-bearing status at baseline and non-inversion injury mechanisms were associated with lower function at 4 months (p<0.01; adjusted R square=0.20). Pain on medial palpation and pain on dorsiflexion at 4 weeks were the most valuable prognostic indicators of function at 4 months (*p*< 0.01; adjusted R square=0.49).

**Conclusion:**

The results of the present study provide further evidence that ankle sprains have a variable clinical course. Age, injury grade, mechanism and weight-bearing status at baseline provide some prognostic information for short and medium term recovery. Clinical assessment variables at 4 weeks were the strongest predictors of recovery, explaining 50% of the variance in ankle function at 4 months. Further prospective research is required to highlight the factors that best inform the expected convalescent period, and risk of recurrence.

## Introduction

Ankle sprains have been reported to constitute 7-10% of all admissions to Accident and Emergency departments [1]. In the United States there are 2.15 ankle sprains per 1000 person-years [2], incurring a significant annual health-care cost. Ankle sprains are often perceived as minor injuries but have a highly variable prognosis. It is estimated that 36-85% of patients report full recovery, with re-injury rates of between 30 and 40% [3]. Serious long term implications associated with ankle sprain include persistent pain, instability, recurrent injury and post traumatic arthritis [3,4].

Most primary care facilities provide advice on controlling acute inflammatory symptoms after ankle sprains [5]. This is part of a ‘one-size fits all’ approach where there is little emphasis on identifying risk factors of poor prognosis at the individual patient level. Other areas of musculoskeletal management have adopted a stepped care approach whereby patients most at risk of poor outcome are identified, and prioritised for early secondary prevention [6].

Key prognostic variables have been identified for other common lower limb injuries. Greater age [7], restricted ankle dorsiflexion [8,9] and pain [9] are all independent predictors of recovery after bony ankle injury. Greater age, clinical grade, length of the muscle tear and a stretching trauma mechanism predict poor outcome after hamstring strain [10]. There is also consistent evidence that greater body mass is associated with poorer recovery after common musculoskeletal injuries including: chronic knee pain [11], anterior cruciate ligament surgery [12], and medial tibial stress injury [13].

Current clinical practice guidelines offer few recommendations on prognostic factors associated with ankle sprain [14]. Consequently, discharge criteria after ankle sprain are often vague and avoid prognostication relating to recovery. Determining important markers of prognosis will provide higher level evidence for clinical decision making and help highlight patients most at risk of inadequate recovery. The primary aim of this study was to identify predictors of short and medium term functional recovery after acute ankle sprain. Our secondary aim was to examine whether the timing of clinical assessment (baseline vs four weeks post injury) affects the prognostic value of common clinical tests.

## Materials and Methods

### Study design

We undertook a secondary analysis of data, obtained from a randomised clinical trial in which a large number of clinical factors were studied. Study protocols have been detailed previously [15]. The randomised controlled study examined the effects of early rehabilitation exercises compared to standard advice (ice, compression and elevation) during the first week after acute ankle sprain. Early rehabilitation exercise had a positive short term effect on the primary outcome (subjective function); this was significant at weeks 1 and 2 post injury. However this study found no between group differences reported at 4 week and 4 month follow up [15].

## Ethics Statement

The Office Regional Ethics Committee NI approved the study and all participants signed a letter of informed consent. Study participants were aged 16-65. For all patients under the age of 18, written informed consent was obtained from their next of kin, caretakers, or guardians.

### Participants

N=101 patients aged 16-65 years with an acute (<7 days) ankle sprain were recruited from an Accident and Emergency department (Royal Victoria Hospital, Belfast) or sports injury clinic (University of Ulster) by two researchers (CMB, SRO’C) Patients were excluded from the study if they presented with gross instability (mechanical instability diagnosed by a positive anterior drawer or inversion stress test) [16], or bony ankle injury (indicated by Ottawa ankle rules [17] or plain x ray films).

### Clinical outcomes and potential predictive variables

The primary outcome measure was ankle function measured using the Karlsson score [18] which was recorded at 4 weeks and 4 months post injury. Variables recorded at baseline and at 4 weeks post injury were investigated for their prognostic ability. To comply with recommendations of at least 10 events per variable investigated [19], the number of predictor variables was restricted to 10. We selected candidate predictor variables considered to be associated with a poor outcome based on previous literature [7–14] and group consensus. The candidate predictor variables included participant characteristics (age, body mass index (BMI), gender); injury variables (injury mechanism, previous injury); injury variables based on clinical examination (injury grade [20], weight-bearing status, medial joint line pain on palpation, weight-bearing dorsiflexion [21] and side hop test [22]) and functional status (Karlsson scores at baseline and at week 4 were also used to determine predictors of Karlsson scores at week 4 and week 16 respectively). Continuous variables were not categorised. The focus of the weight-bearing dorsiflexion [21] and side hop tests [22] was the presence or absence of pain.

### Statistical Analysis

All statistical analyses were performed using SPSS V.17.0 (SPSS Inc, Chicago, Illinois, USA). Baseline data for each predictor variable were presented as means and standard deviations for continuous data, and numbers and percentages for categorical data. Initially we conducted a series of univariate analyses to determine whether any predictor variables were significantly associated with the dependent variable of ankle function (Karlsson score). Correlations among predictor variables were calculated to screen for any strong co-linearity (r>0.8).

Independent variables demonstrating a p value less than 0.10 on univariate testing were included for further regression analysis. A linear multiple regression analysis using a forward step-wise method was used to determine the most important predictors of Karlsson scores at week 4 and week 16 (P<0.01). The strength of the predictive ability of identified factors in each multivariate model was determined using regression coefficients (β), with 95% CI.

## Results

### Overview of study participants and drop out

Table 1 provides a summary of participant characteristics at baseline. Eighty-five participants (84%) were available at final follow-up assessment at 4 months. In all cases, the reasons for drop out were that the participant failed to attend and could not be contacted by phone. Participants had a mean age of 27.0 years (SD 9.8; range 16-58) with a mean BMI of 25 kg/m^2^ (SD 3.18). 70% of participants were male, with the majority suffering an inversion type sprain. All participants were assessed within the first week of injury, the mean time between injury and recruitment in the study was 40 hrs (SD 36 hrs). Almost 50% of participants reported a history of previous ankle injury. Subjective ankle function improved between baseline (mean 48.9/100, SD 19.8, range 15-95), 4 weeks (mean 93.0/100, SD 7.9, range 57-100) and 4 months post injury (mean 97.3/100, SD 4.1, range 80-100).

**Table 1:** Summary baseline characteristics.

Age (yrs)	26.94 (SD 9.78)
BMI (kg/m2)	25.34 (SD 3.18)
Gender (male/female)	69/31
Injury mechanism (Inversion/Other)	67/33
Injury grade (1, 2, 2+)	26/63/11
Medial joint line pain (Y/N)	60/40
Weight-bearing status (FWB; FWB with pain; PWB; NWB)	11/40/36/13
Pain during WB’ing Ankle D/F (Y/N)	80/20
Previous ankle injury (Y/N)	47/53
Baseline Karlsson score (/100)	48.97 (SD 19.8)

FWB, full weight bearing; PWB, partial weight bearing; NWB, non-weight bearing; D/F, dorsiflexion

Means (SD) are presented for continuous data; % are presented for binary/categorical data.

### Colinearity

There were no strong inter-correlations between the independent variables in all combinations, r<0.8.

Thus, all listed independent predictors were included in the univariate analysis.

### Baseline assessment

A number of the predictor variables recorded at baseline (BMI, gender, previous injury, mechanism of injury, medial joint line pain, weight-bearing dorsiflexion, Karlsson scores) were not significantly correlated (P>0.1) with ankle function at week 4 or at week 16 and were not included in the multivariable analyses. The side hop test [22] was deemed to be too provocative for the acute phases of injury and was not assessed at baseline.

Age, grade of injury and weight-bearing status at baseline were univariately correlated (p<0.1) with functional status at week 4. These variables were included in a multivariate analysis; using the stepwise method, a significant model emerged (*F*3,77=14.814, *p* < 0.01) with an adjusted R square value of 0.341) (Table 2).

**Table 2:** Baseline Prognostic Factors for Short Term Recovery (Karlsson score).

**Predictor variable**	**Unstandardised β (95% CI)**	**P value**	**Standardised β**
Age	-0.284 (-0.445 to -0.123)	0.001	-0.319
Grade of injury	-4.369 (-7.154 to -1.584)	0.003	-0.340
Weight-bearing status	-2.145 (-4.166 to -.123)	0.038	-0.229

Age, weight-bearing status and injury mechanism were univariately correlated (p<0.1) with functional status at 4 months and were included in a multivariate analysis (Table 3). Using the stepwise method, a significant model emerged (*F*3,80=7.911, *p* < 0.01) with an adjusted R square value of 0.20.

**Table 3:** Baseline Prognostic Factors Medium Term Recovery (Karlsson score).

**Predictor variable**	**Unstandardised β (95% CI)**	**P value**	**Standardised β**
Age	-0.115 (-0.201 to -0.028)	0.01	-0.262
Weight-bearing status	-1.118 (-2.095 to -0.142)	0.25	-0.228
Injury Mechanism	-2.098 (-3.804 to -0.392)	0.17	-0.245

### Week 4 assessment

A number of the predictor variables recorded at week 4 post injury (weight-bearing status, side hop test and Karlsson score) were not significantly correlated (p>0.1) with ankle function at 4 months, and were not included in the multivariate analysis. Pain during weight-bearing ankle dorsiflexion and medial joint line pain at week 4 were associated with lower functional status at 4 months (Table 4). Using the stepwise method, a significant model emerged (*F*2,75=37.368, *p* < 0.01) with an adjusted R square = 0.49.

**Table 4:** Week 4 Prognostic Factors for Medium Term Function (Karlsson score).

**Variable**	**Unstandardised β (95% CI)**	**P value**	**Standardised β**
Medial joint line pain	4.920 (1.397 to 8.444)	0.07	0.238
Pain on WB’ing Ankle D/F	6.804 (4.858 to 8.751)	0.00	0.597

## Discussion

This study was a secondary analysis on data recorded from participants presenting to primary care clinics with acute ankle sprain. We found further evidence of the variable course of ankle sprain recovery. Age and weight-bearing status on assessment provided useful prognostic information for short and medium term functional recovery. Injury grade and mechanism of injury were also useful predictors of short and medium term function respectively. Physical examination at 4 weeks held most prognostic value and explained 50% of the variation in medium term function (4 months post injury).

### Baseline predictors

Age, weight-bearing status and injury grade explained just over one third of the variation in short term functional recovery. Increasing age seems to be an important predictor of poor recovery after bony [7] and musculoskeletal injury [10]. We also found that participants who were older had a worse prognosis for both short and medium term recovery after ankle sprain. Age-related loss of muscle mass and strength are well documented and may influence the speed and quality of recovery post injury. Other factors such as body weight and physical activity are closely related to age and may impact on recovery. In contrast to our data, previous research found higher BMI to be predictive of poor recovery after a range of musculoskeletal injuries [11–13].

Grading injury severity and weight-bearing ability are key components of acute ankle assessment. We found that participants with higher grades of injury and inability to weight-bear in the early stages had a worse short term prognosis. Although we anticipated significant collinearity between weight-bearing status and injury grade, these predictors were only weakly correlated. This seems to suggest that additional factors influence weight-bearing status. Psychosocial factors such as pain related fear of movement (kinesiophobia) have been shown to negatively impact recovery from chronic musculoskeletal conditions [23]. An interesting finding was that early weight-bearing was also associated with better medium term function. The average time between injury and assessment of weight-bearing status was 40 hours; we found only 50% of participants were able to fully weight-bear (either with or without pain) at this time. There are few guidelines on how patients should progress from early protection to progressive weight-bearing after ankle sprain. There is increasing evidence that early controlled loading of healing tissue is an important determinant of recovery [24–28]. Further research should ascertain whether there is an optimal window for loading tissues after injury.

### Week 4 Predictors

A recent prospective study [29] found that early assessment of ankle sprain could not predict long term outcome. Although we were able to derive some prognostic value from baseline assessment, the richest prognostic information was generated from variables measured at 4 weeks post injury. Specifically the presence of pain during two clinical tests (medial palpation and weight-bearing dorsiflexion [21]) was associated with significantly lower ankle function at medium term follow up.

Clinical guidelines suggest that normal range of movement (ROM) should be achieved within two weeks after ankle sprain [30]. Failure to meet this milestone could be indicative of concomitant injury. Soft tissue impingement, synovitis [31] or altered arthokinematics [32] may cause long term deficits in ROM after ankle sprain. Failure to regain normal ankle kinematics after injury may be an important risk factor for chronic symptoms including recurrent injury [33] and chronic ankle instability [34]. There is some evidence that manual treatment techniques enhance early restoration of ankle dorsiflexion after ankle sprain [35] but the long term effectiveness is unclear.

Medial pain on palpation at week 4 was associated with lower functional status at 4 months. We can only postulate the reasons for persistent pain within our sample. Medial bone bruising can often result after a lateral ankle sprain, usually due to compressive forces. Bone bruises are characterised by a subchondral osseous contusion, and may remain evident on MRI for 6 months after injury [36]. Other potential mechanisms of medial pain include bony impingement between the medial malleolus and the medial facet of the talus due to changes in lateral stability [37]. There is also evidence for increased medial loading of the ankle joint in subjects with ankle instability [38,39]. More serious causes of medial pain include occult joint injury, particularly osteochondral lesions of the talus. Arthroscopic examination of patients with persistent ankle pain (>2 months) highlighted a 40% incidence of osteochondral lesion, of which 28% were missed with physical examination and imaging [40].

Patients with ankle sprain presenting to primary care are rarely examined beyond the acute phases after injury. When initial physical examination is affected by acute pain and swelling, current best practice is to offer reassessment after 3–5 days [16]. Our data suggest that undertaking an additional physical examination at four weeks post injury will offer additional prognostic guidance. Although this would be associated with additional cost and practitioner time, it may allow early identification of patients at risk of persistent disabling symptoms. These patients could then benefit from specialist referral and may be less likely to succumb to chronic joint pain and recurrent injury.

### Limitations and Future study

This study was based on data from 85 participants with acute ankle sprains and represents one of the largest study cohorts based on prognostic indicators. We acknowledge that a retrospective analysis of RCTs is not the most effective method to evaluate prognosis. However data were collected prospectively therefore recall bias is not an issue. Future prospective studies with sample size powered for prognostic analysis and a priori selection of predictor variables are required. The best selection model for entering independent variables into multivariate analyses is controversial. We used a forward stepwise selection model; limitations associated with this approach include sampling error and inflated risk of Type 1 error. These limitations are less relevant to the current study as we ensured that the number candidate predictor variables were comparable to our sample size [41].

The current evidence base provides a poor guide for clinicians who are planning treatment and predicting recovery in the acute phases after ankle sprain [14]. Further research is needed to develop prognostic models in the primary care setting. Family doctors and Accident and Emergency practitioners often have little time to assess musculoskeletal injuries therefore the key is to develop simple prognostic models [6]. We have provided some evidence towards a refined clinical examination model. Clinicians should be mindful of patients with ankle sprain presenting with persistent pain on medial palpation and painful active dorsiflexion. Additional follow up in the sub-acute phases should be considered and could facilitate more accurate prognostication of longer term function.
